# Outcomes of Valve-in-Valve (VIV) Transcatheter Aortic Valve Replacement (TAVR) after Surgical Aortic Valve Replacement with Sutureless Surgical Aortic Valve Prostheses Perceval™: A Systematic Review of Published Cases

**DOI:** 10.3390/jcm13175164

**Published:** 2024-08-30

**Authors:** Tamer Owais, Osama Bisht, Mostafa Hossam El Din Moawad, Mohammad El-Garhy, Sina Stock, Evaldas Girdauskas, Thomas Kuntze, Mohamed Amer, Philipp Lauten

**Affiliations:** 1 Department of Cardiac Surgery, University Hospital Augsburg, 86156 Augsburg, Germany; tamerowaiss1976@yahoo.com (T.O.); sina.stock@uk-augsburg.de (S.S.); herzchirurgie@uk-augsburg.de (E.G.); 2 Department of Cardiothoracic Surgery, Cairo University, Giza 12163, Egypt; 3 Department of Cardiology and Angiology, Regiomed Klinikum Coburg, 96450 Coburg, Germany; obisht@gmail.com; 4 Department of Clinical Pharmacy, Faculty of Pharmacy, Alexandria 21513, Egypt; mh3912214@gmail.com; 5 Department of Cardiology, Heart Vascular Center, 36199 Rotenburg an der Fulda, Germany; 6 Heart Center, Zentralklinik Bad Berka, 99437 Bad Berka, Germany; kac@zentralklinik.de (T.K.); philipp.lauten@zentralklinik.de (P.L.); 7 Department of Cardiac Surgery, Heart Centre Siegburg-Wuppertal, University Witten-Herdecke, 42103 Wuppertal, Germany; mohamedishaq2000@yahoo.com

**Keywords:** TAVI, TAVR, valve-in-valve, perceval

## Abstract

**Background**: Valve-in-Valve (VIV) transcatheter aortic valve replacement (TAVR) is a potential solution for malfunctioning surgical aortic valve prostheses, though limited data exist for its use in Perceval valves. **Methods**: searches were performed on PubMed and Scopus up to 31 July 2023, focusing on case reports and series addressing VIV replacement for degenerated Perceval bioprostheses. **Results**: Our analysis included 57 patients from 27 case reports and 6 case series. Most patients (68.4%) were women, with a mean age of 76 ± 4.4 years and a mean STS score of 6.1 ± 4.3%. Follow-up averaged 9.8 ± 8.9 months, the mean gradient reduction was 15 ± 5.9 mmHg at discharge and 13 ± 4.2 mmHg at follow-up. Complications occurred in 15.7% of patients, including atrioventricular block III in four patients (7%), major bleeding or vascular complications in two patients (3.5%), an annular rupture in two patients (3.5%), and mortality in two patients (3.5%). No coronary obstruction was reported. Balloon-expanding valves were used in 61.4% of patients, predominantly the Sapien model. In the self-expanding group (38.6%), no valve migration occurred, with a permanent pacemaker implantation rate of 9%, compared to 5.7% for balloon-expanding valves. **Conclusions**: VIV-TAVR using both balloon-expanding and self-expanding technologies is feasible after the implantation of Perceval valves; however, it should be performed by experienced operators with experience both in TAVR and VIV procedures.

## 1. Background

Surgical aortic valve replacement (AVR) is the recommended treatment option for patients with aortic valve disease [1,2]. The operational risk for AVR has reduced significantly, with a drop in mortality from 4.3% to 2.6% [3,4]. Even with these results, elderly high-risk patients who are referred for AVR still have poor outcomes; hence, sutureless technology may assist in reducing morbidity and mortality [5,6,7,8].

A whole new approach to surgically implanting aortic valve bioprostheses has been described in the last ten years with the advent of sutureless aortic valve replacement (SAVR). The Perceval valve (LivaNova, PLC, London, UK) is a well-known sutureless bioprosthesis that has been demonstrated to cut the cross-clamp time in half [7,9]. The Perceval valve differs from traditional surgical sutured bioprostheses in that it features a tall nitinol stent composed of two rings joined by nine struts. Regarding sutured bioprostheses, sutureless bioprostheses are susceptible to structural valve degeneration; reports of stent infolding, maybe as a result of improper prosthetic sizing technique, have also been made [7]. Initially, older patients were the primary target market for SAVR operations, as they stand to gain the most from shorter recovery times. Patients in this higher risk category, however, will be at much greater danger should the prior sutureless bioprosthesis degenerate and require surgery. Given the encouraging outcomes of recent series, a percutaneous technique using Valve-in-Valve (VIV) surgery could be taken into consideration as a viable alternative in this case [10]. Transcatheter aortic valve replacement (TAVR) is a viable method for the VIV replacement of surgical valves that have deteriorated. Nonetheless, there is still little experience with VIV-TAVR for deteriorated Perceval valves [11,12,13]. Since, there are only published cases in the literature explaining the outcomes of TAVR through VIV procedure, we aimed to gather evidence from all available cases to generate a more comprehensive view of this procedure.

## 2. Methods

This study was carried out in compliance with the Cochrane Handbook of Systematic Reviews of Interventions at each step [14]. Following the PRISMA statement’s guidelines, we conducted this systematic review and meta-analysis [15].

### 2.1. Data Sources and Search Strategy

**Figure 1:**
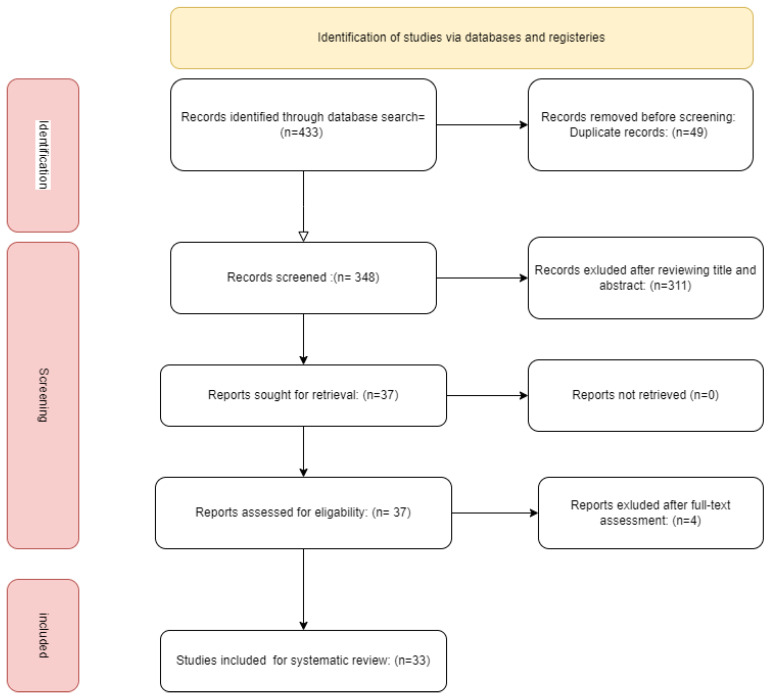
flow chart of the study.

The searching process was conducted on PubMed, Scopus, and Web of Science from inception to 30 June 2024Since they better illustrate the purpose of our study, the following keywords were included in the search strategy: “Perceval” OR “sutureless valve” AND “Aortic valve replacement” OR “AVR” AND “Valve in Valve” (Figure 1).

### 2.2. Eligibility Criteria

All papers were examined separately by four authors during the selection and critical appraisal phases. Case reports and case series investigating the use of VIV replacement for degenerated Perceval bioprostheses were included in our systematic review. Narrative reviews, scoping reviews, systematic reviews, conference abstracts, randomized controlled trials, descriptive cross-sectional studies, case-control studies, and cohort studies were all excluded because they did not address the key goals. Rayyan [16] was used to screen the articles based on their titles and abstracts. Each prospective article’s whole text was reviewed individually by four authors after title and abstract screening. Any disagreements that developed throughout the research selection process were resolved by consensus and if the disagreements persisted, the senior author resolved them.

### 2.3. Data Extraction

Four authors worked separately to extract data using a standard data extraction sheet created in Microsoft Excel and individually updated by the senior author. The following data were extracted: Age, sex, STS Scor%, Perceval size, cause, reason for VIV, degeneration, type of TAVI, duration of follow-up, mean gradient at discharge, and, on follow-up, complications, coronary obstruction, and outcomes.

### 2.4. Quality Assessment

The Joanna Briggs Institute Critical Appraisal tools for Case Reports were used to evaluate the quality of the included studies [17]. The questions were answered with “Yes” or “No”. Each “yes” response is worth one point, and the possible scores range from 0 to 8. Studies with a score of 7–8 are of good quality, those with a score of 4–6 are of a moderate level, and those with a score of 0–3 are of low quality. Only publications with a score more than four were included in this study.

## 3. Results

### 3.1. Database Searching and Screening

According to our abovementioned search and quality assessment criteria, we included 48 patients in this metanalysis from 27 case reports and 5 case series [18,19,20,21,22,23,24,25,26,27,28,29,30,31,32,33,34,35,36,37,38,39,40,41].

### 3.2. Baseline Characteristics

The majority (68.4) were women and the mean ± SD of age was 76 ± 4.4 years. The STS score % of the included patients had a mean ± SD of 6.1 ± 4.3% and the months after Perceval implant were 9.8 ± 8.9 months. The mean TAVI size was 24.7 ± 1.9 mm, while the Perceval size was small in 31.5% of the patients, medium in 40.3%, large in 22.8%, and x-large in 5.2%. The most common reason for VIV was steno-insufficiency in 45.6% of patients, followed by stenosis and regurgitation in 31.5% and 22.8% of patients, respectively. The most common mechanism was degeneration (36 patients; 63.1%), followed by stent folding (13 patients; 22.8%); otherwise, migration in three patients, thrombosis in two patients, and endocarditis in one patient. The majority (61.4%) of implanted TAVI was balloon expanding and the most common model (57.8%) was Sapien. (Table 1)

In the patients treated with self-expanding valves (20 patients; 41.6%), including 17 patients with CoreValve or Evolut, 1 patient with Acurate neo, 1 patient with Portico, and 1 patient with Allegra, no valve migration was reported, and the rate of permanent pacemaker (PPM) implantation was 10%, compared to 7.1% in patients treated with balloon-expanding technology.

### 3.3. Clinical Outcomes

The mean follow-up period was 9.8 ± 8.9 months; the mean gradient reduction was 15 ± 5.9 mmHg at discharge and 13 ± 4.2 mmHg at follow-up. The follow-up data were available in 19 patients; all those patients were asymptomatic or had improved symptoms at the follow-up. Complications were reported in nine patients (15.7%). Among these, four patients (7%) developed atrioventricular (AV) block III, with one patient experiencing late AV block III on the 8th postoperative day. Major bleeding or vascular complications were reported in two patients (3.5%), an annular rupture in two patients (3.5%), and mortality in two patients (3.5%). The majority of patients (84.3%) showed no complications. The cause of mortality was an annular rupture in one patient, while the second patient underwent an emergency ViV TAVR to treat severe aortic regurgitation (AR) and cardiogenic shock due to Perceval migration. This patient died 10 days after the procedure due to complications derived from the mechanical support implanted the day after the procedure. Moreover, none of the patients showed coronary obstruction. (Table 2) Balloon-expanding valves were used in 61.4% of patients, predominantly the Sapien model. In the self-expanding group (38.6%), no valve migration occurred, with a permanent pacemaker implantation rate of 9%, compared to 5.7% for balloon-expanding valves.

Two cases suffered from annular ruptures; the first had post-procedural imaging which demonstrated bioprosthetic valve frame protrusion and contained an annular rupture, which required operative intervention. The second had an aortic annular rupture inferior to the origin of the left coronary artery with the extravasation of contrast and a large hematoma compressing the right ventricular outflow tract. Upon a review of previous imaging, the rupture site appeared to correspond to the location of the previously infolded portion of the Perceval valve. The patient passed away on the second postoperative day (Table 3 and Table 4).

## 4. Discussion

Our study aimed to evaluate the outcomes of VIV-TAVR in patients with Perceval implants. We observed that VIV-TAVR, utilizing both balloon-expanding and self-expanding TAVR technologies, demonstrated safety and efficacy, yielding excellent short- and mid-term outcomes and favorable hemodynamics.

Both surgical and transcatheter bioprosthetic valves have a limited lifetime. It is often challenging to identify patients with failed bioprosthetic valves as historical definitions are based on death or valvular reinterventions, meaning that all cases are not accurately captured. Thus, the accurate capture of all valve failure cases should be based on imaging. The main modality for diagnosis is echocardiography and, to a lesser extent, computerized tomography which can add valuable information regarding the mechanism. The echocardiographic definition of structural valve degeneration includes an increase in the mean transvalvular gradient ≥10 mm Hg resulting in a mean gradient ≥20 mm Hg with concomitant decreases in the aortic valve which are (AVA) ≥0.3 cm^−2^ or ≥25% and/or decreases in the dimensionless velocity index (DVI) ≥0.1 or ≥20% compared with the echocardiographic assessment performed 1-to-3 months postprocedure or at discharge if not available or new occurrence or an increase of >1 grade of intraprosthetic aortic regurgitation (AR) resulting in moderate AR [47]. The mechanisms of bioprosthetic valve dysfunction include structural bioprosthetic valve failure, i.e., intrinsic irreversible damage to the valve structure; non-structural valve failure, i.e., not intrinsic to the valve; valve thrombosis; and endocarditis [47]. Examples of non-structural valve degeneration include a patient–prosthesis mismatch and paravalvular regurgitation.

Current ESC guidelines recommend operative Re-SAVR for patients with degenerated bioprosthetic valves as a class I-C recommendation. However, many patients with degenerated valves have operative risk, thus the guidelines recommend TAVR as the treatment of choice after a multidisciplinary heart-team discussion and risk assessment using standardized scores like STS or EuroScores [48].

The “PERCEVAL TRIAL” pilot study was performed in 30 high-risk patients who were scheduled for isolated SAVR due to severe aortic stenosis, the Perceval met non-inferiority criteria vs. stented AV-prosthesis, but had a significantly reduced surgical times mean (CPB: 71.0 ± 34.1 vs. 87.8 ± 33.9 min; mean aortic cross-clamp times: 48.5 ± 24.7 vs. 65.2 ± 23.6; both p-values < 0.001), but resulted in a higher rate of permanent pacemaker implantation (PPI—11.1 vs. 3.6% at 1 year). Incidences of paravalvular leakage (PVL) and central leak were similar [49]. Another study reported excellent outcomes with Perceval with a total of 1652 patients who showed excellent short-term and long-term outcomes [8]. A recent study by Concistrè et al. [50] showed that VIV-TAVR seems to be a safe and efficient solution for deteriorated Perceval. This process is linked to a superior hemodynamic function and positive clinical outcomes.

The majority of patients in our study were elderly patients and had a high surgical risk with a mean STS-Score ± SD of 6.1 ± 4.3%; thus a transcatheter treatment was recommended over reoperation, denoting a high degree of guidelines-based treatment in the study cohort.

The most commonly degenerated valves were small and medium valves, and the most common mode of failure was mixed stenosis and regurgitation. Valve degeneration is often multifactorial and cannot be attributed to a single factor. In a recent study including a total of 25,490 patients, risk factors included an increasing body surface area, a patient–prosthesis mismatch, and smoking, whereas age was a protective factor [51]. In our study, mixed stenosis and regurgitation was the most prevalent type of degeneration. The degeneration of bioprothesis is a complex multifactorial process; however, some points should be specifically avoided when implanting Perceval. Many surgeons favor some degree of oversizing in conventional SAVR, thus achieving superior hemodynamics and avoiding a patient–prosthesis mismatch. However, this should not be carried out in cases of sutureless rapid-deployment Perceval because it leads to the incomplete expansion and or deformation of the stent struts, causing regurgitation or stenosis. This was proven by Cerillo et al. [52], who found that the degree of oversizing is the strongest predictor of high postoperative peak and mean gradients. Thus, it is felt that the incorrect sizing of the Perceval may be the culprit in early valve degeneration.

Regarding the choice of the TAVI prosthesis, we found that both the balloon-expanding and self-expanding prosthesis were successfully deployed with no difference in the outcome noted. We found that the peak and mean gradients met the VARC-3 definition for device success without increased gradients in 100% of the patients.

In our series, no cases of coronary artery obstruction occurred. This highlights the importance of preoperative planning. Commonly used measures include the virtual transcatheter heart valve (THV) to the coronary ostial distance (VTC), which can be simulated prior to implantation. Thus, a VTC of less than 3 mm is considered a high risk for coronary obstruction, and 6 mm is considered a low risk [53]. Our hypothesis is that the configuration of PERCEVAL has a crucial role in preventing iatrogenic coronary obstruction. This valve has a flask-shaped suprannular frame, which prevents the displacement of the degenerated leaflet towards the sinus of Valsalva, providing adequate space between the coronary ostia and the degenerated bioprosthesis. This allows for adequate coronary perfusion by preserving the VTC. Concerning the pacemaker after the valve-in–valve-out analysis shows an incidence of 0.0% in comparison with TAVR in sutured bioprosthetics. This could be explained by the relative flexibility of the PERCEVAL frame compared to the stiff sewing ring on the sutured counterpart. This flexibility may allow the overexpansion in PERCEVAL and hence injury to the conduction system with a higher incidence of conduction disturbance and permanent pacemakers. One useful technique is to assess the depth of the in situ Perceval and its relationship to the membranous septum, thus avoiding this potential caveat. This also could have implications for the choice of TAVR valve, depth of implantation, and overexpansion and may be avoidable in the hands of experienced teams.

Regarding the hemodynamic performance of the implanted TAVI prosthesis, it is well known that the ViV-TAVI prosthesis implanted in the surgical valve has a poorer hemodynamic profile with elevated mean gradients and a lower-than-expected orifice area when compared to Redo-SAVR [54]. This may be an important issue regarding the lifetime management of younger patients undergoing ViV-TAVR after SAVR. In our study, most of the patients achieved a low mean gradient, with the mean gradient decreasing to 11 mmHg on the 6-month follow-up. This superior hemodynamic performance after Perceval may be attributed to the overexpansion capacity of the valve without the need for valve fracture. This is attributed to the flexible Nitinol–Titanium alloy allowing successful overexpansion, thus achieving superior hemodynamics.

## 5. Limitations

One limitation of our meta-analysis is the relatively small number of patients included, which may affect the generalizability of our findings, in addition to the lack of long-term follow-up and the absence of follow-up echocardiographic data from 71% of the patients.

## 6. Conclusions

ViV-TAVR, utilizing both balloon-expanding and self-expanding TAVR technologies, demonstrated acceptable outcomes. Although it remains a relatively rare procedure, it can be performed safely in the hands of an experienced TAVR operator after the appropriate discussion of all treatment options by the heart team. Experience with the VIV procedure after sutureless valves remains limited and should be further studied to provide a consensus on the management of those patients.

## Figures and Tables

**Table 1:** Demographic and baseline characteristics of the included patients.

Characteristics		Mean	Standard Deviation (SD)
Age		76	4.4
STS score %		6.1	4.3
Months after Perceval implant		9.8	8.9
TAVI size (mm)		24.7	1.9
		Number	%
Gender	Males	18	31.5%
	Female	39	68.4%
Perceval size	Small	18	31.5%
	Medium	23	40.3%
	Large	13	22.8%
	X-large	3	5.2%
Reason for ViV	Stenosis	18	31.57%
	Regurgitation	13	22.8%
	Steno-insufficiency	26	45.6%
Cause	Degeneration	36	63.1%
	Stent folding	13	22.8%
	Migration	3	5.2%
	Cusp thrombosis	2	3.5%
	Endocarditis	1	1.7%
TAVI implanted	Balloon-expanding	35	61.4%
	Self-expanding	22	38.6%
TAVI model	Sapien	33	57.8%
	Evolut/Core	19	33.3%
	Myval	2	3.5%
	Others (Accurate, Portico, Allegra)	3	5.2%

SD: standard deviation, STS: society of thoracic surgeon, TAVI: transcatheter aortic valve implantation, ViV: Valve in Valve.

**Table 2:** Clinical characteristics of the included patients.

Characteristic		Mean	Standard Deviation
Follow-up (months)		9.8	8.9
Mean gradients at discharge (mmHg)		15	5.9
Mean gradients at follow-up (mmHg)		13	4.2
		Number	Percentage
Status at follow-up, *n* = 16	Asymptomatic or improved symptoms	16	100%
	With symptoms	0	0%
Complications	Yes	9	15.7%
	No	38	84.3%
Coronary obstruction, n = 45	Yes	0	0%
	No	57	100%

*n*: number.

**Table 3:** Procedural characteristics of the included patients.

N	Author	Year	Age (yrs)	G	STS %	P Size	Days after P Implant	Reason for ViV	Cause	TAVI Model	TAVI Size (mm)	mPG	FU (m)	mPG FU (mmHg)	Comp.
1	Sun	2018	71	M	16.4	S	21	steno-insufficiency	Stent folding	Edwards SAPIEN 3	26	9.7	6	9.1	no
2	Lettieri	2017	84	F		M	1825	steno-insufficiency	Degeneration and stent folding	Corevalve Evolute R	26				no
3	Mangner	2018	75	F	8.1	S	1825	steno-insufficiency	Degeneration, regurgitation, and stenosis	Edwards SAPIEN 3	23	21			no
5	Amabile	2015	78	F		S	1095	regurgitation	Failure	Edwards SAPIEN 3	23		1		no
6	Di Eusanio	2015	80	F		M	5	regurgitation	Stent folding	Edwards SAPIEN XT	23				no
7	Karla	2018	75	F		M	21	steno-insufficiency	Stent folding	Edwards SAPIEN 3	20				no
9	Landes	2018	80	F		S	730	steno-insufficiency	Stent folding	Edwards SAPIEN XT	23	10	6		no
10	Fujita	2015	77	F	4.2	S	1095	steno-insufficiency	Degeneration	Edwards SAPIEN XT	23	8	2		no
11	Amabile	2016	83.2	F	9.6	M	1144.45	steno-insufficiency	Degeneration	Evolut R	26	23	3	10	no
11	Amabile	2016	82.9	F	7.2	M	2425.87	stenosis	Degeneration	Evolut R	26	33	3	20	no
11	Amabile	2016	83.8	F	6	M	2212.81	steno-insufficiency	Degeneration	Evolut R	26	16	3	8	no
11	Amabile	2016	80.9	M	4.7	L	1999.74	stenosis	Degeneration	Evolut R	26	16	3	19	no
11	Amabile	2016	72.4	F	16.3	M	1905.39	steno-insufficiency	Degeneration	Corevalve	23	32	3	15	no
12	Belluschi	2021	67	M		L	2920	regurgitation	Failure	Myval	26	10	1	10	no
13	Vondran	2021	85	F	11.9	M	1095	steno-insufficiency	Degeneration	Allegra	23	9			no
14	Misfeld	2020	77	F		S	2920	stenosis	Degeneration	Sapien 3	20	24	12	13	no
14	Misfeld	2020	82	F		L	2190	stenosis	Degeneration	Evolut R	29	11	3	6	no
14	Misfeld	2020	76	F		S	2190	steno-insufficiency	Degeneration	Sapien 3	23	23	3	17	bleeding into the right groin
14	Misfeld	2020	85	F		S	1460	stenosis	Degeneration	Sapien 3	20		3	17	no
15	Ellouze	2020	79	F		M	1095	steno-insufficiency	Degeneration	CoreValve	26	9	8		no
15	Ellouze	2020	84	F		S	1460	steno-insufficiency	Degeneration	Sapien	23	7	6		no
15	Ellouze	2020	86	M		L	1461	stenosis	Degeneration	Sapien	26	10	1		AV block with PPI, 8 days post-TAVR
17	Vilalta	2020				S	350	stenosis	Stent folding	Evolut PRO	23	27	6	15	AV block III
17	Vilalta	2020				S	1139	steno-insufficiency	Stent folding	Acurate Neo	23	19	6	17	AV block III
17	Vilalta	2020				S	1249	steno-insufficiency	Degeneration	Sapien 3	23	16	6	10	no
17	Vilalta	2020				M	1811	steno-insufficiency	Degeneration	Sapien 3	23	10	6	7	no
17	Vilalta	2020				XL	978	regurgitation	Failure	Sapien 3	26	9	6	7	no
18	Raschpichler	2019	81	F		M	213	regurgitation	Stent folding	Sapien 3	23	10	6		no
19	Morales-Portano	2019	76	M	4	M	1	regurgitation	Dislocation	Corevalve	29	10			no
20	Laricchia	2019	73	M		M	0.25	regurgitation	Partial migration	Evolut PRO	29		1		no
21	Kosmas	2019	71	F		S	2191	steno-insufficiency	Degeneration	Evolut PRO	23	35.8			no
22	Koni	2019	88	F		L	2555	steno-insufficiency	Degeneration	Sapien 3 Ultra	26	16			no
23	Belluschi	2019	74	M	2	XL	365	steno-insufficiency	Stent deformation	Evolut R	29		12		no
24	Balghith	2019	70	F		M	1825	steno-insufficiency	Degeneration	Sapien 3	26	15			no
25	Garcia-Lara	2018	79	F	4.3	S	1826	stenosis	Degeneration	Sapien 3	23	10	2		AV block III
26	Oezpeker	2018	77	F	10.01	M	304	cusps thrombosis	Cusp thrombosis	Sapien 3	23	10	3	10	no
27	Suleiman	2021	78	F		M	1917	regurgitation	Degeneration	Sapien 3	23	13,2			Vasc. access pouch stented
27	Suleiman	2021	72	F		S	1339	regurgitation	Degeneration	CoreValve	23	9	10	9	no
27	Suleiman	2021	73	F		M	1369	stenosis	Degeneration	CoreValve	23	30	3		no
27	Suleiman	2021	66	F		L	2130	stenosis	Degeneration	CoreValve	26	7	4	7	no
28	Tomai	2021	73	M		M	240	steno-insufficiency	Endocarditis leading to degeneration	Sapien 3	23 mm	16			no
29	Medda	2020	84	F	17,4	L	2190	steno-insufficiency	Malfunction	Abbott Portico	25				no
30	Loforte	2022	83	F		M	3	steno-insufficiency	Stent folding	Sapien	23		48		no
31	López-Tejero	2022	67	F	1,02	S	0.25	regurgitation and occlusion ofthe left main coronary artery	Migration	Evolut Pro	26				death
32	Kay Robert T.	2022	75	F		M	1095	stenosis	Stent folding	SAPIEN 3	23				annular rupture and death
33	Patterson T.	2020	81	F		S	730	regurgitation	Stent folding	SAPIEN 3	23	NA			annular rupture
34	Erdogan	2022	70	F	4,5	L	1826	stenosis	Degeneration	Myval	26	7			no
35	Arslan	2021	70	M		M	93	regurgitation	Stent folding and paravalvular failure jet	SAPIEN 3	23	11			no
36	Dubois	2023	74	F		L	1825	regurgitation	Degeneration	sapien 3	26	13			no
36	Dubois	2023	83	F		L	2217	stenosis	Degeneration	Sapien 3	23	18			no
36	Dubois	2023	75	f		L	1715	stenosis	Degeneration	Sapien 3	23	17			no
36	Dubois	2023	82	M		M	1314	stenosis	Degeneration	Sapien 3	23	12			no
36	Dubois	2023	76	M		L	2774	stenosis	Degeneration	Sapien	26	14			no
36	Dubois	2023	80	F		L	2372	stenoso-insufficency	Degeneration	Evolut R	26	11			no
36	Dubois	2023	78	M		M	1788	steno-insufficiency	Degeneration	Sapien 3	23	28			no
36	Dubois	2023	79	F		S	1643	steno-insufficiency	Degeneration	sapien 3	23	8			no
36	Dubois	2023	82	M		XL	2226	stenosis	Degeneration	Evolut R	29	17			no

N: number, yrs: years, G: gender, STS: society of thoracic surgeon, P: perceval, mPG: mean pressure gradient, m: months, FU: follow-up, comp.: complications.

**Table 4:** Outcomes of Valve in Valve procedure in the included patients.

Study ID	Outcomes
Kay et al., 2022 [11].	TTE on the same day showed a well-seated SAPIEN valve, with normal gradients and no regurgitation. However, a significant compression of the right ventricular outflow tract from an extrinsic source was seen, which was not evident on previous imaging. An emergent gated CT angiogram of the chest revealed an aortic annular rupture inferior to the origin of the left coronary artery with extravasation of contrast and a large hematoma compressing the right ventricular outflow tract. Upon review of previous imaging, the rupture site appeared to correspond to the location of the previous infolded portion of the Perceval valve. The patient was stable through the day, with sudden deterioration in the early morning, when she passed away.
Suleiman et al., 2022 [12]	The patient was discharged successfully two days after the procedure and is clinically much improved, and no gradient/regurgitation.
Suleiman et al., 2022 [12]	The patient tolerated the procedure well with no gradient or regurgitant jet apparent across the Perceval CoreValve combination. Owing to the acuity and complexity of the patient’s condition, their total hospital stay was 25 days. However, she was discharged home 2 days following ViV-TAVI and was asymptomatic from a cardiac standpoint when seen in clinic 6 months post-procedure. An echocardiogram 10 months post-procedure showed a well-seated valve with no regurgitation apparent and a MG of 9 mmHg.
Suleiman et al., 2022 [12]	From an aortic valve standpoint, the Perceval-CoreValve apparatus remained well seated on echocardiogram 3 months post-procedure with no regurgitant jet. There was a higher than expected peak velocity of 3.7 m·s^−1^ without apparent valve leaflet dysfunction and this has been attributed to effective patient prosthesis mismatch, anemia, and associated hyperdynamic circulation.
Suleiman et al., 2022 [12]	Good hemodynamic effect was demonstrated with minimal gradient across the valve. The patient had an uneventful recovery and was discharged day 1 post-procedure. Echocardiography 4 months post-procedure showed an MG of 7 mmHg and no para-valvular leak.
Belluschi, et al., 2021 [13]	Excellent angiographic results, with mild AR. No complications. After uneventful in-hospital stay, the patient was discharged 4 days later, maintaining similar echo parameters at 30-day follow-up.
Sun et al., 2018 [18]	No complications, excellent angiographic results, proper valve-in-valve function with no significant gradients or regurgitation, and uncomplicated postoperative course. Patient remained asymptomatic at 6 months.
Lettieri et al., 2017 [19]	No complications. The pre-discharge multidetector CT showed the correct positioning of the distal margin of the Evolut R approximately 2 mm above the distal ring of the sutureless valve, with minimal compression of the transcatheter valve at that level, but with circular shape of the perimeter in correspondence to the Evolotu R leaflets. The patient was discharged and at 90 days, the postoperative course was uneventful.
Mangner et al., 2018 [20]	No complications. After ViV there was an immediate improvement of the regurgitation and of the stenosis. Echo predischarge showed no regurgitation, a mean gradient of 21 mmHg and an aortic valve area of 1.3 cm^2^, indicating a moderate patient prosthesis mismatch already existing directly after first operation.
Durand et al., 2015 [21]	No complications. A final supra-aortic angiogram showed no residual aortic regurgitation and hemodynamic status improved rapidly. The clinical status of the patient improved dramatically with rapid normalization of liver and renal function tests within 72 h. Clinical course was uneventful and the patient was discharged home 5 days later. At 30-day follow-up, the patient was asymptomatic with return to normal life.
Di Eusanio, et al., 2015 [22]	No complications. The TAVI was successfully deployed with excellent angiographic results. The post-procedural course was uneventful; renal function improved and TTE at discharge showed no significant leaks and gradients across the TAVI implanted.
Fujita et al., 2015 [23]	No complications occurred.
Amabile et al., 2016 [24]	The success rate was 100%. There was no device migration, neither death or periprocedural adverse events, and no need for a second device in any patient. A mild post-procedural aortic regurgitation was noted in 2 patients. The 30-day post-procedural clinical course was uneventful in all subjects. Mean transaortic gradient significantly decreased over time: median mean transprosthetic gradient was 45 ± 26 mmHg before procedure, 24 ± 16 mmHg immediately after, and 14 ± 9 Hg mm 30 days after TAVI (*p* < 0.05 vs. baseline for D0 and D30, *p* < 0.05 for D30 vs. D0, t Student paired test). Comparable results were observed at 90 days.
Vondran et al., 2021 [25]	No postdilatation was needed in view of the excellent hemodynamic results with an invasive transvalvular gradient of 5 mmHg. Based on the Valve Academic Research Consortium II criteria, no major adverse event occurred during the hospital stay. The patient was discharged 9 days after the procedure to a rehabilitation center with a maximum/mean transvalvular pressure gradient measured by transthoracic echocardiography of 19/9 mm Hg and no apparent paravalvular leakage.
Misfeld et al., 2020 [26]	The patient left the hybrid operation room in a hemodynamically stable condition. Postoperative transthoracic echocardiography (TTE) demonstrated normal LV function and a mean gradient across the valve of 24 mmHg. Effective orifice area was calculated at 1.0 cm^2^. There was only trivial paravalvular leak. The patient was discharged on postoperative day two. At 12-month follow-up, the patient showed improvement in clinical symptoms (NYHA I–II). Pressure gradients had further decreased (mean gradient 13 mmHg) and the effective orifice area was measured at 1.2 cm^2^. There was only trivial paravalvular leakage.
Misfeld et al., 2020 [26]	The postoperative course was uneventful. On TTE, LV function had slightly improved (LV ejection fraction 47%). Mean pressure gradient was 11 mmHg and effective orifice area was calculated at 1.8 cm^2^. There was mild residual aortic regurgitation. The patient was discharged on postoperative day three. At 3-month follow-up, clinical symptoms had improved (NYHA I–II°). TTE showed an LV ejection fraction of 48% and a low mean pressure gradient of max 6 mmHg with an effective orifice area of 1.9 cm^2^. There was still mild aortic regurgitation.
Misfeld et al., 2020 [26]	Analysis of the cerebral protection system revealed large debris in both filters. The early course was complicated by bleeding into the right groin, which was treated conservatively. On TTE, LV ejection fraction was 47%, mean gradient was slightly increased (23 mmHg), and the effective orifice area was 1.1 cm^2^. There was only trivial residual aortic regurgitation. The patient was discharged on POD five. At 3-month follow-up, this patient had also clinically improved (NYHA I–II). LV function also improved (LV ejection fraction 57%) and mean gradient was 17 mmHg with an effective orifice area of 1.5 cm^2^. There was only trivial residual aortic regurgitation.
Misfeld et al., 2020 [26]	Analysis of the cerebral protection system revealed debris in both filters. The early course was uncomplicated and the patient was discharged on postoperative day 3. At 3-month follow-up, this patient had also clinically improved, but still had dyspnea (NYHA II–III). LV function was normal and mean gradient across the aortic valve prosthesis was 17 mmHg with an effective orifice area of 1.2 cm^2^. There was no residual aortic regurgitation. During the last admission, an Amplatzer Vascular Plug III (St. Jude Medical Inc., MN, USA) was implanted to close the residual gap in the LAA.
Ellouze et al., 2020 [27]	(a) ViV transcatheter aortic valve implantation in sutureless valves was feasible and safe; (b) challenging cases such as small degenerated valves were successfully treated with self-expanding valves and acceptable gradients, (c) the rate of procedural complications was low and good in-hospital and mid-term outcomes were acheived with different types of transcatheter aortic valves.
Vilalta et al., 2020 [28]	NR
Raschpichler et al., 2019 [29]	At 6-month follow-up, no change respecting the prosthesis implanted was observed, with CT showing full expansion of the surgical valve frame, circular geometry of the ViV complex, and no evidence of frame recoil
Morales-Portano et al., 2019 [30]	Successful with no complications.
Laricchia et al., 2019 [31]	No complications. Trivial residual AR. The patient was discharged on the sixth postoperative day in good general conditions. After 1 month, he repeated a CT scan demonstrating good positioning and shaping of the ViV.
Kosmas et al., 2019 [32]	NR
Koni et al., 2019 [33]	Good hemodynamic results without leak and uneventful hospitalization. No complications
Balghith et al., 2019 [34]	Excellent final results, no PVL and the gradient was 15 mmHg. The patient was discharged from hospital after 2 days in a very good condition. No complications
Garcia-Lara et al., 2018 [35]	(After implantation): The hemodynamic outcome was optimal with a resulting maximum gradient of 20 mmHg and a mean gradient of 10 mmHg, with no significant regurgitation. Angiography showed that the prosthesis remained in a position slightly below the lower edge of the Perceval prosthesis. After implantation, the patient experienced complete atrioventricular block. A provisional pacemaker was implanted with implantation of a definitive VVIR device after 48 h. The patient progressed favorably and was discharged 5 days after implantation. Two months later, she remained asymptomatic.
Oezpeker et al., 2018 [36]	No complications. Post-operative echocardiography 5 days and 6 and 12 weeks after TAVI showed regular AV function with a constant minimal MPG (≤10 mmHg) and excluded valvular regurgitation. CT scan on Day 5 after TAVI confirmed perfect anatomical positioning of the AV with a completely circular shape of the annulus [diameter 20.4 × 20.1 mm]. The course of NT-proBNP levels was 2260 ng/L before initial aortic valve replacement, 2892 ng/L before the ViV procedure, and 1475 ng/L immediately thereafter. The calculated systolic PAP decreased from 40 mmHg before ViV-TAVI to 30 mmHg. The patient presented with NYHA I 1 month after ViV-TAVI.
Tomai et al., 2021 [37]	Angiography and echo-color-Doppler evaluation both indicated the absence of aortic regurgitation or paravalvular leaks. The patient was discharged 5 days later, in good clinical conditions. The so-called Matryoshka procedure, that is, a valve-in-valve-in-valve TAVI procedure with a balloon-expanding Sapien 3 valve following a surgical valve-in-valve procedure with a suture-less bioprosthesis implanted into a degenerated stented aortic valve, is safe and feasible (operative mean gradient).
López-Tejero et al., 2022 [38]	Neither regurgitation nor significant gradient was detected after implantation. After the initial recovery, the patient died 10 days later due to complications derived from the mechanical support implanted the day after the procedure.
Patterson et al., 2020 [39]	Post-procedural imaging demonstrated bioprosthetic valve frame protrusion and contained annular rupture, which required operative intervention (Level of Difficulty: Intermediate).
Erdogan et al., 2022 [40]	There was no significant gradient and/or paravalvular regurgitation on control aortic root angiography after THV implantation, and both the coronary arteries were patent.
Arslan et al., 2021 [41]	Symptoms of heart failure started during the follow-up, which suggests that the stent-infolding of the valve might occur in the late postoperative period.
Kalra, et al., 2018 [42]	There was complete resolution of transaoortic gradient and restoration of circular valve conformation, and no complications occurred.
Landes, et al., 2018 [43]	The TAVI was successfully deployed with excellent angiographic results. The rest of the hospitalization course was uneventful. At discharge, echocardiography showed proper ViV functioning with no significant leaks or gradients across the Sapien XT valve. The patient remained asymptomatic for the following 6 months.
Loforte et al., 2022 [44]	She presented type III AV block on the third day and progressive worsening of hemodynamics and respiratory function. The echocardiogram revealed the presence of moderate aortic regurgitation and high pressure gradients through the implanted valve (max/mean gradients: 52/31 mmHg; prosthetic valve area: 1.15 cm^2^) that appeared to be “heart shaped” as a result of a partial collapse of the basal ring at the level of the right coronary sinus; this structural distortion jailed the right coronary cusp and caused moderate-to-severe aortic regurgitation. Urgent transcatheter balloon valvuloplasty was successfully performed to reshape the valve and to stabilize the hemodynamics, but the patients developed the same imaging and clinical pattern 9 days after.
Medda et al., 2020 [45]	Good results were obtained without complications.
Dubois et al., 2023 [46]	Good results were obtained without complications.

## Abbreviations

AR	Aortic Regurgitation
AV	Atrioventricular
AVR	Aortic Valve Replacement
CPB	Cardiopulmonary Bypass
ESC	European Society of Cardiology
PPM	Permanent Pacemaker
PRISMA	Preferred Reporting Items for Systematic Reviews and Meta-Analyses
SAVR	Sutureless Aortic Valve Replacement
STS	Society of Thoracic Surgeons
TAVR	Transcatheter Aortic Valve Replacement
THV	Transcatheter Heart Valve
VARC-3	Valve Academic Research Consortium 3
VIV	Valve-in-Valve
